# The systemic oxidative stress score has a prognostic value on gastric cancer patients undergoing surgery

**DOI:** 10.3389/fonc.2024.1307662

**Published:** 2024-03-08

**Authors:** Xinyu Wang, Limin Zhang

**Affiliations:** Department of Gastrointestinal Surgery, Harbin Medical University Cancer Hospital, Harbin Medical University, Harbin, Heilongjiang, China

**Keywords:** oxidative stress, SOS, surgery, prognosis, gastric cancer

## Abstract

**Background:**

Oxidative stress is strongly associated with the development, recurrence metastasis, and treatment of gastric cancer. It is yet unknown, though, how systemic oxidative stress levels relate to the surgically treated gastric cancer patients’ clinical results. This research aims to investigate the prognostic effect of systemic oxidative stress score, also known as systematic oxidative stress score (SOS), on gastric cancer patients undergoing surgical treatment.

**Methods:**

Development of the SOS Formula through Least Absolute Shrinkage and Selection Operator LASSO Cox Regression. By using optimal cut-off values, the 466 patients included in the study had been split into high SOS and low SOS groups. Utilizing Chi-square test and the Wilcoxon rank sum test, this research examined the relationship between SOS and clinical traits. With the aid of Kaplan-Meier and COX regression analysis, the prognosis of patients with gastric cancer was examined.

**Results:**

SOS consisted of four oxidative stress-related laboratory indices. Univariate and multivariate COX regression analyses revealed that SOS, Age, CA724, Radical resection and TNM stage were crucial prognostic factors for OS, and the independent prognostic factors for PFS included Age, CA724, TNM stage and SOS. They could have their prognosis correctly predicted using a nomogram built around SOS and independent prognostic variables.

**Conclusion:**

SOS is a practical and reasonably priced tool for determining a patient’s prognosis for gastric cancer. More notably, SOS is an accurate prognostic factor for patients with advanced gastric cancer who has undergone radical surgery.

## Introduction

The highest incidence of gastric cancer in the world is still found in East Asia, despite the fact that the mortality rate has been decreasing due to the development of a variety of cancer treatment modalities and the growing popularity of screening for Helicobacter pylori, a major risk factor for gastric cancer (1–4). Although the survival rates for gastric cancer in China have significantly increased since, 2000, the disease still poses a serious threat to the country’s public health (5).

The clinical results of patients with late gastric cancer and distant metastases are still a matter for concern, despite the fact that the adoption of immunotherapy and targeted therapy has improved patients’ survival (6–8). Although stage and severity of patients’ disease do have an impact on survival time, and the prognosis of gastric cancer, nutrition and inflammatory status also have a substantial effect on patients’ prognosis and course of therapy (9, 10).

For instance, cancer patients frequently experience cachexia and weight loss. Cachexia is a condition that causes individuals to lose weight while also losing skeletal muscle and adipose tissue (11). It is caused by a combination of enhanced wasting capacity, abnormally high catabolism, and inflammation. The patient’s life treatment, follow-up care, and length of survival are impacted by this (12). Chronic inflammation is a significant contributor to tumor growth and has a beneficial effect on the tumor micro-environment by encouraging the formation of tumor blood vessels and lymphatic vessels as well as boosting metastasis and tumor dissemination (13). The chronic inflammation mentioned above and cachexia are both closely related to oxidative stress. Studies have shown that oxidative stress can encourage protein catabolism by activating the nuclear factor-kb (NFkB) molecular mechanism and initiating the ubiquitin-proteasome pathway in skeletal muscle, both of which reduce the amount of protein in the muscle (14, 15). Patients with cachexia cancer experience increased oxidative stress due to decreased intake of nutrients (including antioxidants), altered metabolism impairing the production of reducing compounds, and increased ROS brought on by chronic inflammation’s overproduction of pro-inflammatory cytokines (16). In conclusion, oxidative stress influences patient prognosis and is a factor in cachexia development. The transcription factors NF-B, AP-1, p53, HIF-1, PPAR-, -linked protein/Wnt, and Nrf2 are also activated by persistent oxidative stress, which is the root cause of chronic inflammation. The expression of several inflammatory genes, such as IL-1, IL-6, and IL-8, which are produced by pro-inflammatory cells, is therefore increased as a result of these transcription factors (17). Oxidative stress is a significant factor in cancer.

Greater emphasis should be paid to the oxidative stress status of gastric cancer patients. From the perspective of the epidemiology causing gastric cancer, diet, smoking, and H. pylori infection can all lead to an imbalance in oxidative stress status (18, 19). Also, in terms of gastric cancer treatment, increased levels of oxidative stress might hasten the development of drug resistance to particular treatments in patients with gastric cancer (20). One example of this is the part that oxidative stress plays in the mechanism of oxaliplatin resistance (21–23). Consequently, treatment with medicines like cisplatin might increase oxidative stress levels in patients (24). What’s more to that, surgery, the most common treatment option for individuals with gastric cancer, generates a substantial number of oxygen and nitrogen radicals. The preoperative SOS in surgically treated patients was the focus of this study because of its prognostic potential.

## Methods

### Research population

This study retrospectively included surgically treated 466 patients with gastric cancer, 413 of whom underwent radical gastrectomy for gastric cancer. They all underwent surgery in, 2016-2022 at the Cancer Hospital of Harbin Medical University. Their detailed clinical information can be accessed through an electronic case system which archive patients’ clinical characteristics, blood biochemical indicators, tumor markers, and pathological staging.

Patients eligible for inclusion in the study were those whose pathology supported a diagnosis of gastric cancer, those who underwent surgical treatment and those without severe cardiovascular or psychiatric disease. Patients without clinical data or unable to undergo surgical treatment were excluded. The standards of the Declaration of Helsinki and its subsequrevisions were adhered to in this study, which was reviewed by the Institutional Review Board (IRB) and approved by the Harbin Medical University Oncology Affiliation. (Ethics number: 2019-57-IIT).

### Follow-up

PFS and OS of the study subjects were obtained by telephone follow-up after data collection. The follow-up included: laboratory tests (routine blood, liver and kidney function, tumor markers), imaging (computed tomography, supraclavicular ultrasound); follow-up period: stage I: every 12 months; stage II: 6 months; stage III: 3 months; stage IV, recurrence: anytime. The term “progression-free survival” (PFS) refers to the period of time between the date of surgery and the date of disease progression, metastasis, or death, or the date of the final follow-up. The time from the date of operation until decease or the final check-up was known as overall survival (OS).

### Statistical methods

Receiver operating characteristic (ROC) curves were used for the optimal cut-off values of SOS and its related laboratory indicators. The Wilcoxon rank sum test was used to compare variances between two groups for continuous variables that had a positive-terrestrial distribution. Continuous variables that did not have this distribution were marked as median and interquartile spacing. The chi-square test was applied to compare two groups of qualitative data, which were expressed as the percentage of cases (%). The Kaplan-Meier method along with the Log-rank test were conducted to calculate the survival curves for OS and PFS. With the use of Cox regression analysis, both univariate and multivariate, independent prognostic variables of patients were found. In multivariate Cox regression analysis, univariate Cox regression analysis P<0.05 was taken into account, and relative risk was assessed using HR and 95% confidence intervals. SPSS 25.0 (SPSS Inc., Chicago, IL, USA) and R 4.2.3 (Vienna, Austria) were applied to build all statistical analyses, with P<0.05 considered a meaningful variance between the two groups.

## Result

### Creation of SOS

**Figure 1:**
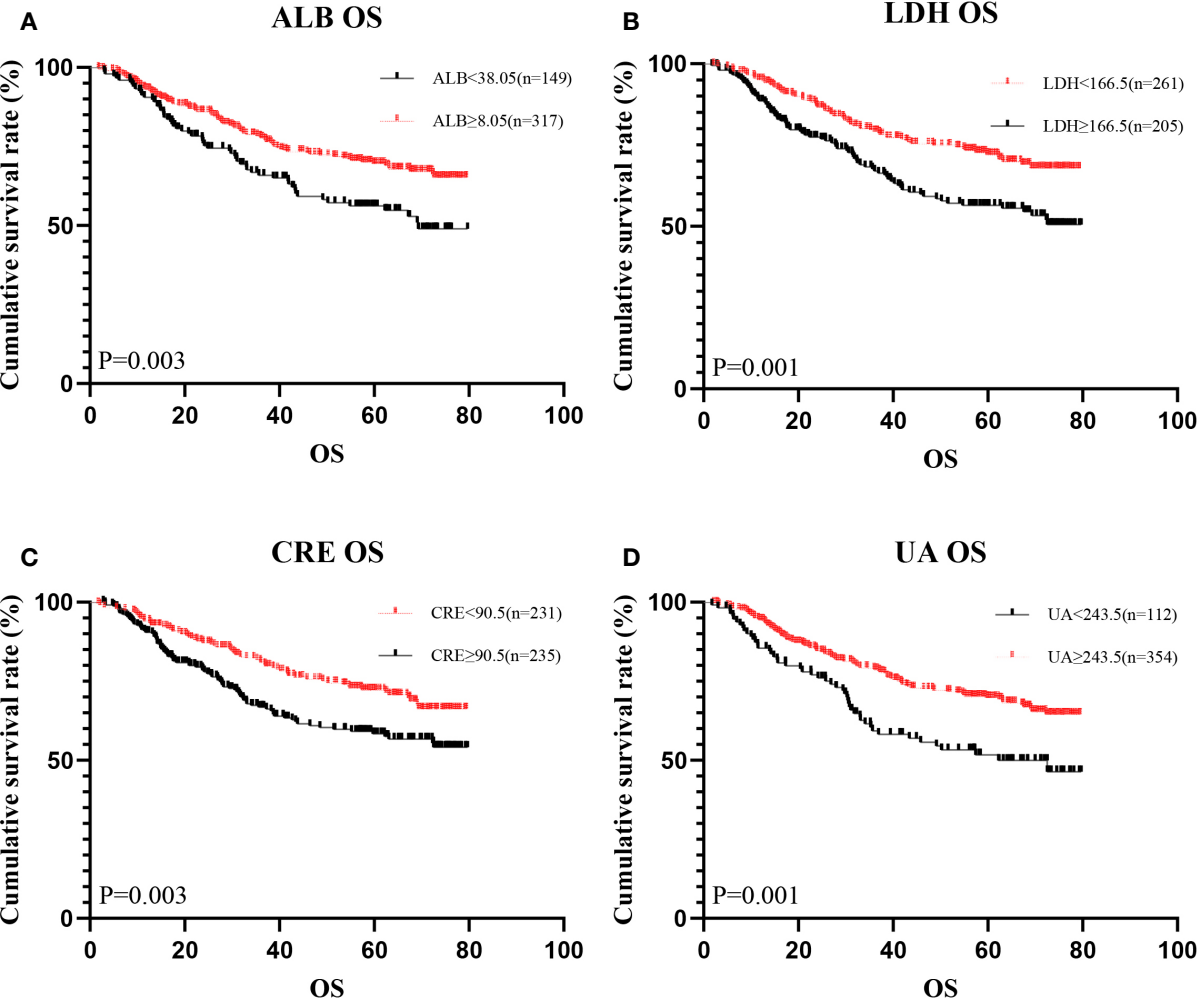
Kaplan-Meier analysis showing survival curve for OS of oxidative stress biomarker. #Kaplan–Meier curves for overall survival (OS), stratified by **(A)** ALB, **(B)** LDH, **(C)** CRE, **(D)** UA.

**Figure 2:**
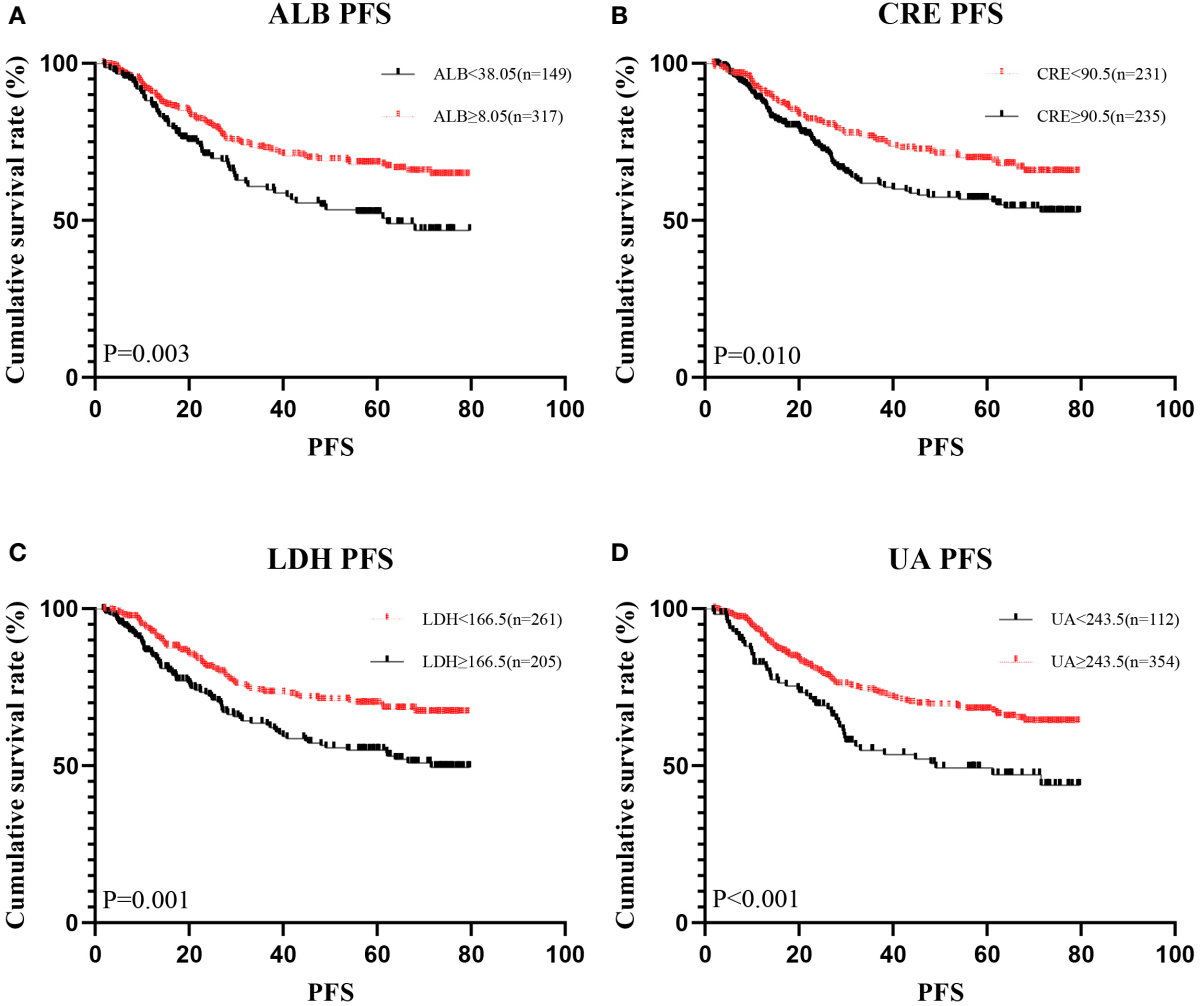
Kaplan-Meier analysis showing survival curve for PFS of oxidative stress biomarker. # Kaplan–Meier curves for progression-free survival (PFS), stratified by **(A)** ALB, **(B)** LDH, **(C)** CRE, **(D)** UA.

**Figure 3:**
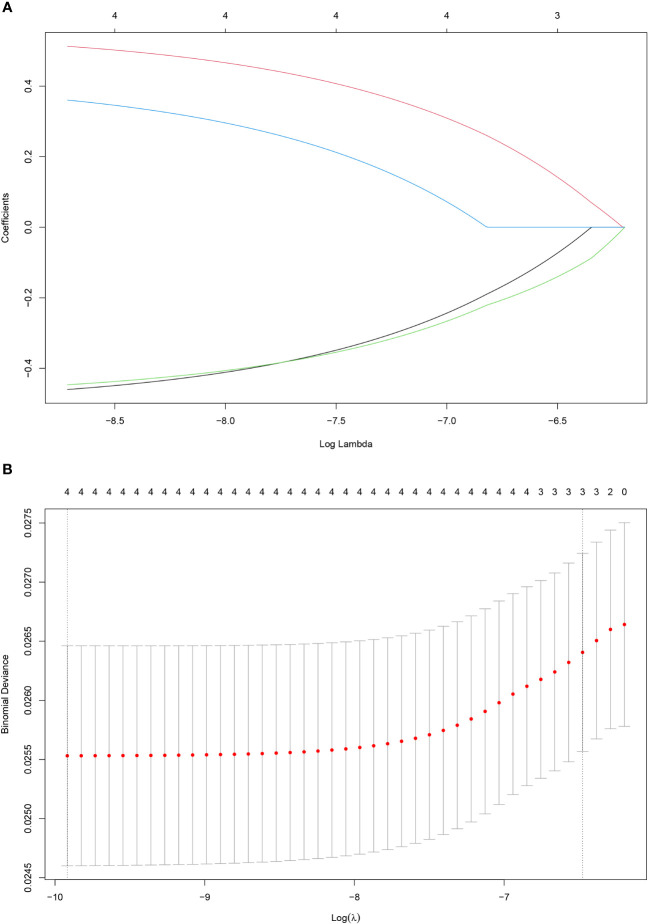
Construction of the SOS using the LASSO Cox regression model. Bias difference of LASSO coefficient profiles **(A)**. Least Absolute Shrinkage and Selection Operator (LASSO) coefficient profiles **(B)** for 4 oxidative stress-related biomarkers.

A total of 466 patients, with a mean age of 58.22 years, was enrolled in this investigation, of which 88.6% underwent radical surgery. The baseline demographic and clinical features of the patients, segregated into training (n = 326) and validation cohorts (n = 140), are detailed in **Table 1**. To assess the prognostic implications of systemic oxidative stress indices, these indices were dichotomized using critical values determined via ROC analysis. Kaplan-Meier survival analysis was conducted for oxidative stress-related biochemical indices, with those exhibiting P<0.05 then subjected to LASSO Cox regression analysis. Consequently, a Systemic Oxidative Stress (SOS) score was derived from variables with non-zero coefficients, where low albumin (ALB) and uric acid (UA) levels correlated with poorer OS and PFS, while high lactate dehydrogenase (LDH) and creatinine (CRE) levels were associated with adverse OS and PFS outcomes (all p<0.05) (**Figures 1**, **2**). The SOS formula was established as SOS = 0.554 * LDH + 0.404 * CRE - 0.493 * ALB - 0.474 * UA, and its optimal cut-off value determined by ROC was -9.21 (**Figure 3**).

**Table 1:** Clinicopathological characteristics.

Variables	All patients(n=466)	Training cohort(n=326)	Validation cohort(n=140)
Age (year), median(SD)	58.22 (10.38)	58.47 (9.75)	57.49 (11.67)
Sex(%)			
male	325 (69.7)	237 (72.7)	88 (62.9)
Female	141 (30.3)	89 (27.3)	52 (37.1)
BMI (Kg/m2), mean (SD)	22.51 (3.23)	24.07 (29.87)	22.55 (3.57)
Radical resection (%)			
Yes	413 (88.6)	286 (87.7)	127 (90.7)
No	53 (11.4)	40 (12.3)	13 (9.3)
Primary tumor site (%)			
Upper 1/3	23 (4.9)	14 (4.3)	9 (6.4)
Middle 1/3	46 (9.9)	30 (9.2)	16 (11.4)
Low 1/3	386(82.8)	277(85.0)	109(77.9)
Whole	11(2.4)	5(1.5)	6(4.3)
Lauren type (%)			
intestinal	176(37.8)	124(38.0)	52(37.1)
diffuse	75(16.1)	50(15.3)	25(19.7)
mixed	127(27.3)	83(25.5)	44(31.4)
unknown	88(18.9)	69(21.2)	19(13.6)
Tumor size (%)			
<20 mm	191(41.0)	128(39.3)	63(45.0)
20-50 mm	172(36.9)	115(35.2)	57(40.7)
>50 mm	103(22.1)	83(25.5)	20(14.3)
Differentiation (%)			
Poor	219(47.0)	158(48.5)	61(43.6)
Moderately	184(39.5)	118(36.2)	66(47.1)
Well	27(5.8)	20(6.1)	7(5.0)
Unknown	36(7.7)	30(9.2)	6(4.3)
TNM stage (%)			
I	132(28.3)	88(27.0)	44(31.4)
II	109(23.4)	76(23.3)	33(23.6)
III	161(34.5)	110(33.7)	51(36.4)
IV	64(13.8)	52(16.0)	13(8.6)
CEA, median (SD)	13.97(81.13)	13.45(82.21)	15.16(78.87)
CA199, median (SD)	48.29(154.60)	49.40(152.96)	43.74(158.83)
CA724, median (SD)	12.39(49.54)	14.70(57.44)	7.08(21.23)
CA125, median (SD)	24.97(117.12)	20.36(65.76)	35.57(188.01)
ALB, median (SD)	40.02(4.29)	40.19(4.28)	39.64(4.32)
LDH, median (SD)	170.13(64.09)	172.10(73.87)	165.16(32.89)
UA, median (SD)	300.86(81.75)	305.34(81.52)	290.56(81.63)
CRE, median (SD)	81.91(30.15)	80.94(15.20)	84.15(49.73)
SOS, median (SD)	-34.78(53.38)	-36.50(55.30)	-31.77(48.67)

#BMI: body mass index; pTNM: pathologic tumor node metastasis; CEA:carcinoembryonic antigen; CA199:carbohydrate antigen 199; CA724:carbohydrate antigen 724; CA125II:carbohydrate antigen 125II.

### The association between SOS and clinical traits and laboratory data

The cohort was stratified based on Systemic Oxidative Stress (SOS) cutoff values, resulting in 141 patients in the high SOS level group and 325 patients in the low SOS level group. The association between SOS levels and various clinical features and tumor markers is elucidated in **Table 2**. Noteworthy differences were observed in clinical characteristics, including Age, Sex, Body Mass Index (BMI), pathological TNM (pTNM) staging, and tumor size across distinct SOS groups. Regarding tumor markers, patients exhibiting high SOS levels displayed a tendency toward elevated Carcinoembryonic Antigen (CEA) levels, alongside reduced levels of Cancer Antigen 199 (CA199) and Cancer Antigen 125 (CA125II) (all p<0.05).

**Table 2:** The association between SOS and clinical traits and tumor marker.

n	level	Low SOS	High SOS	P
		325	141	
Sex	male	268 (75.9)	57 (50.4)	<0.001
	female	85 (24.1)	56 (49.6)	
Age	median (SD)	57.66 (10.22)	59.81 (10.72)	0.031
BMI	median (SD)	22.75(3.03)	26.30 (50.78)	0.001
pTNM	I	107 (30.3)	25 (22.1)	0.041
	II	88 (24.9)	21 (18.6)	
	III	116 (32.9)	45 (39.8)	
	IV	42 (11.9)	22 (19.5)	
Radical resection	R0	317 (89.8)	96 (85.0)	0.158
	non-R0	36(10.2)	17 (15.0)	
Primary Tumor site	upper 1/3	17 (4.8)	6 (5.3)	0.672
	middle 1/3	37 (10.5)	9 (8.0)	
	low 1/3	292 (82.7)	94 (83.2)	
	whole	7(2.0)	4 (3.5)	
Tumor size	<50 mm	161 (45.6)	30 (36.5)	0.001
	≥50 mm	118 (33.4)	54 (47.8)	
	unknown	74 (21.0)	29 (25.7)	
Differentiation	poorly differentiated	163 (46.2)	56 (49.6)	0.318
	moderately differentiated	143 (40.5)	41 (36.3)	
	well differentiated	23 (6.5)	4 (3.5)	
	unknown	24 (6.8)	12 (10.6)	
Lauren type	intestinal	139 (39.4)	37 (32.7)	0.495
	diffuse	56 (15.8)	19 (16.8)	
	mixed	96 (27.2)	31 (27.4)	
	unknown	62 (17.6)	26 (23.1)	
CEA (10^9^ng/mL)	median (SD)	12.37 (79.46)	19.01 (86.38)	0.005
CA199 (U/mL)	median (SD)	28.26 (88.61)	12.50 (111.64)	0.011
CA724 (U/mL)	median (SD)	11.76 (50.14)	14.40 (47.40)	0.091
CA125II (U/mL)	median (SD)	25.25 (129.32)	24.08 (65.42)	0.011

#BMI, body mass index; pTNM, pathologic tumor node metastasis; CEA, carcinoembryonic antigen; CA199, carbohydrate antigen 199; CA724, carbohydrate antigen 724; CA125II, carbohydrate antigen 125II.

### Univariate and multivariate Cox regression analyses

Univariate and multivariate Cox regression models were utilized to analyze the establishment of independent prognostic factors in these patients. The findings demonstrated that SOS was an independent prognostic factor for these patients in training cohorts. Among them, Age (HR = 1.641, P = 0.015), CA724 (HR = 1.962, P = 0.001), Radical resection (HR = 1.769, P = 0.036) and TNM stage (HR = 1.953, P < 0.001) were also independent prognostic factors for OS, and the independent prognostic factors for PFS included Age (HR = 1.578, P = 0.025), CA724 (HR = 2.092, P < 0.001) and TNM stage (HR = 2.112, P < 0.001) as well in **Table 3**.

**Table 3:** Univariate and multivariate Cox regression analyses.

PFS		Training set				Validation set		
	Univariate analysis		Multivariate analysis		Univariate analysis		Multivariate analysis	
Parameters	Hazard ratio (95%CI)	P value	Hazard ratio (95%CI)	P value	Hazard ratio (95%CI)	P value	Hazard ratio (95%CI)	P value
Sex (Male vs Female)	0.848(0.546-1.317)	0.464			1.327(0.716-2.461)	0.369		
Age (<59 vs ≥59)	1.731(1.168-2.564)	0.006	1.579(1.058-2.356)	0.025	1.605(0.854-3.017)	0.142		
CEA (<2.02 ng/mL vs ≥2.02 ng/mL)	1.571(1.071-2.305)	0.021	1.087(.0720-1.641)	0.692	1.358(0.741-2.490)	0.323		
CA199 (<10.49 U/mL vs ≥10.49 U/mL)	1.621(1.106-2.374)	0.013	1.283(0.860-1.915)	0.222	1.0.32(0.563-1.891)	0.920		
CA724 (<2.41 U/mL vs ≥2.41 U/mL)	2.607(1.748-3.887)	<0.001	2.092(1.381-3.168)	<0.001	1.055(0.576-1.932)	0.863		
CA125II (<11.60 U/mL vs ≥11.60 U/mL)	1.810(1.235-2.651)	0.002	1.054(0.689-1.614)	0.808	1.111(0.606-2.036)	0.734		
Tumor size (<5cm vs ≥5cm + Unknown)	2.774(1.808-4.255)	<0.001	0.990(0.591-1.659)	0.970	3.921(1.921-8.003)	<0.001	2.105(0.978-4.259)	0.057
Differentiation (poorly differentiated vs others)	0.837(0.575-1.219)	0.354			0.782(0.425-1.438)	0.428		
Radical resection (R0 vs Non-R0)	3.786(2.361-6.070)	<0.001	1.704(0.995-2.918)	0.052	3.644(1.505-8.820)	0.004	1.973(0.764-5.098)	0.160
TNM stage (I vs II vs III vs IV)	2.544(2.052-3.154)	<0.001	2.112(1.635-2.729)	<0.001	2.926(1.977-4.331)	<0.001	2.379(1.565-3.617)	<0.001
SOS (<-9.21 vs ≥-9.21)	2.85(1.472-3.243)	<0.001	1.709(1.119-2.661)	0.013	1.850(0.983-3.479)	0.052		
OS								
Sex (Male vs Female)	0.829(0.534-1.288)	0.405			1.347(0.727-2.498)	0.344		
Age (<59 vs ≥59)	1.824(1.231-2.703)	0.003	1.641(1.099-2.449)	0.015	1.483(0.789-2.789)	0.021	1.587(0.827-3.004)	0.165
CEA (<2.02 ng/mL vs ≥2.02 ng/mL)	1.744(1.188-2.561)	0.005	1.324(0.877-1.999)	0.181	1.276(0.696-2.337)	0.431		
CA199 (<10.49 U/mL vs ≥10.49 U/mL)	1.701(1.161-2.493)	0.006	1.256(0.841-1.875)	0.266	1.041(0.568-1.908)	0.898		
CA724 (<2.41 U/mL vs ≥2.41 U/mL)	2.563(1.719-3.820)	<0.001	1.962(1.299-2.964)	0.001	1.025(0.559-1.878)	0.937		
CA125II (<11.60 U/mL vs ≥11.60 U/mL)	1.696(1.159-2.483)	0.007	1.001(0.657-1.523)	0.998	1.079(0.589-1.978)	0.805		
Tumor size (<5cm vs ≥5cm + Unknown)	2.604(1.699-3.992)	<0.001	1.012(0.603-1.698)	0.965	3.862(1.888-7.899)	<0.001	1.998(0.906-4.363)	0.087
Differentiation (poorly differentiated vs others)	0.891(0.613-1.296)	0.547			0.794(0.432-1.459)	0.457		
Radical resection (R0 vs Non-R0)	3.739(2.332-5.995)	<0.001	1.769(1.038-3.014)	0.036	3.161(1.303-7.669)	0.011	1.544(0.576-4.136)	0.388
TNM stage (I vs II vs III vs IV)	2.305(1.874-2.834)	<0.001	1.953(1.520-2.511)	<0.001	2.794(1.891-4.130)	<0.001	2.312(1.495-3.575)	<0.001
SOS (<-9.21 vs ≥-9.21)	2.204(1.486-3.270)	<0.001	1.700(1.118-2.586)	0.013	1.964(1.043-3.698)	0.033	1.768(0.927-3.374)	0.084

### Survival analysis

**Figure 4:**
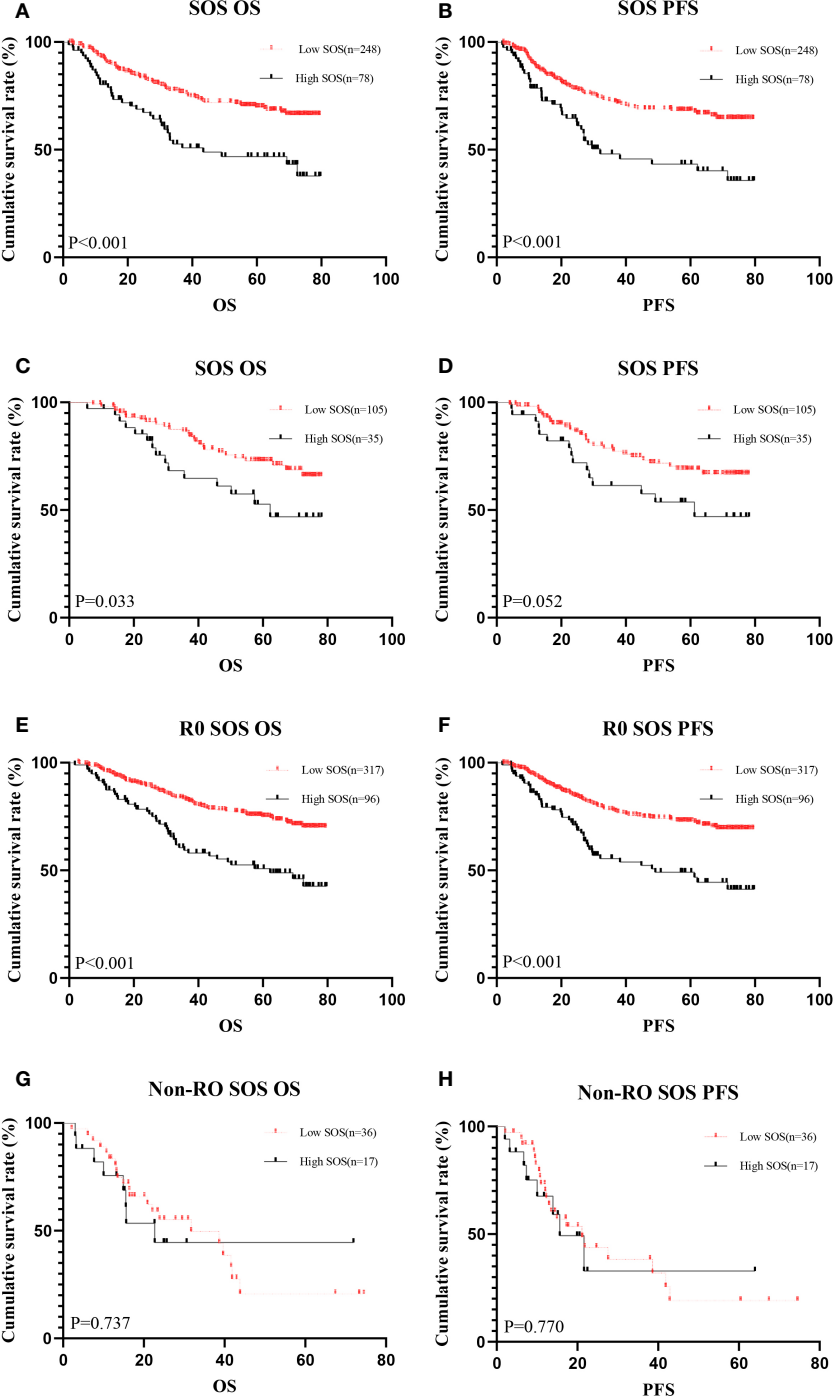
Kaplan-Meier analysis for the PFS and OS of SOS. #Kaplan–Meier curves of OS **(A)** and PFS **(B)** for patients in the low and high groups according to the SOS in the training cohorts. Kaplan–Meier curves of OS **(C)** and PFS **(D)** for patients in the low and high groups according to the SOS in the validation cohorts. Kaplan-Meier analysis showing survival curve for OS **(E)** and PFS **(F)** of patients in R0 subgroup. Kaplan-Meier analysis showing survival curve for OS **(G)** and PFS **(H)** of patients in Non-R0 subgroup.

The prognostic significance of SOS was systematically investigated in gastric cancer patients undergoing surgical treatment, with distinct analyses conducted for both the training and validation cohorts. Within the training cohorts, lower SOS levels were notably associated with enhanced OS (median, not reached vs 43.40 months, P < 0.001) and PFS (median, not reached vs 32.07 months, P < 0.001) than high SOS levels (**Figures 4A, B**). Similarly, in the validation cohorts, lower SOS levels were linked to improved OS (median, not reached vs 62.70 months, P = 0.033) than high SOS levels, albeit without statistically significant predictive value for PFS (median, not reached vs 61.23 months, P = 0.052); nevertheless, the median survival in the low SOS group surpassed that in the high SOS group (**Figures 4C, D**).

In-depth subgroup analyses were performed to discern the impact of SOS within the context of surgical resection outcomes, distinguishing between R0 (complete resection) and Non-R0 (incomplete resection) subgroups. Intriguingly, within the R0 subgroup, SOS exhibited a robust predictive effect (**Figures 4E, F**). However, in the Non-R0 subgroup, SOS failed to attain statistical significance in predicting clinical outcomes (**Figures 4G, H**).

### Nomogram

**Figure 5:**
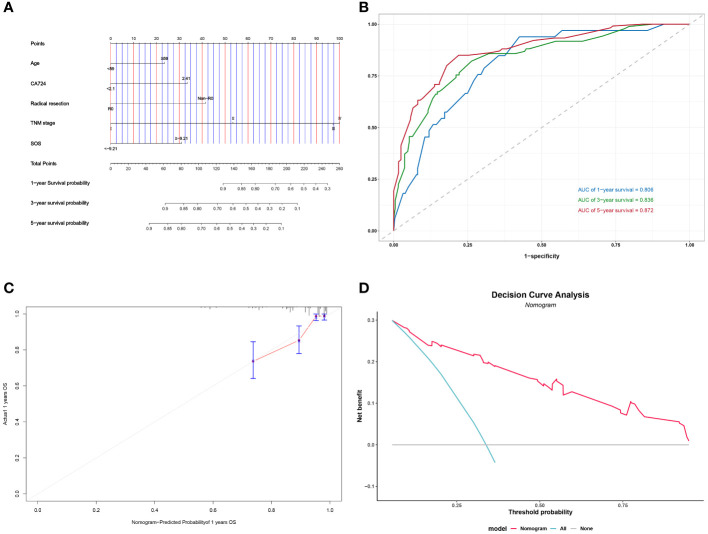
Construction and validation of the nomograms for OS. # Nomogram for OS **(A)**; Time-dependent ROC for nomogram **(B)**; 1-year calibration curve for nomogram **(C)**; Decision Curve Analysis for nomogram **(D)**.

**Figure 6:**
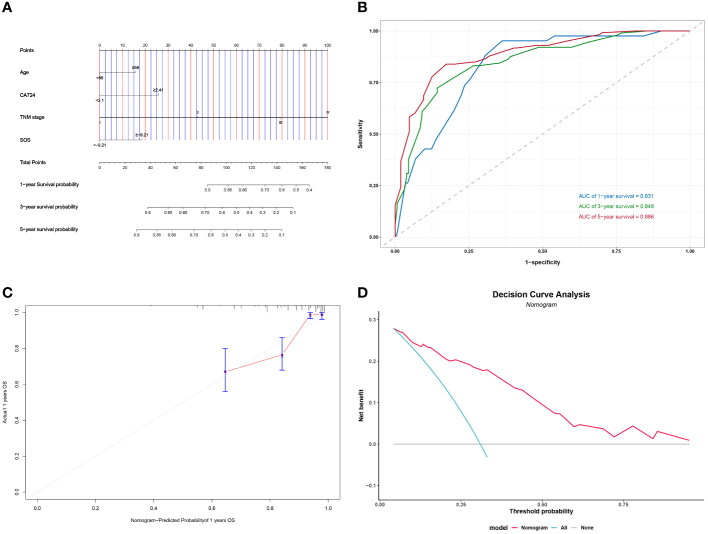
Construction and validation of the nomograms for PFS. # Nomogram for OS **(A)** Time-dependent ROC for nomogram **(B)** 1-year calibration curve for nomogram **(C)** Decision Curve Analysis for nomogram **(D)**.

Following the implementation of multivariate COX analysis to construct prognostic nomograms for both PFS and OS (**Figure 5A** and **Figure 6A**), the derived models exhibited robust predictive performance, as indicated by C-indexes of 0.780 and 0.799 for PFS and OS, respectively. To further assess the accuracy of these models in prognostication, additional analyses were conducted.

Time-dependent ROC curves were plotted, demonstrating impressive predictive capabilities over 1-, 3-, and 5-year intervals for both PFS and OS, with corresponding AUC areas ranging from 0.806 to 0.886 (**Figure 5B** and **Figure 6B**). Calibration curves for the initial year affirmed the nomogram’s precise predictive properties (**Figure 5C** and **Figure 6C**).

Moreover, Decision Curve Analysis (DCA) revealed that the nomogram could provide tangible clinical benefit to patients undergoing gastric cancer surgery (**Figure 5D** and **Figure 6D**). These comprehensive validations underscore the reliability and utility of the prognostic models, highlighting their potential to guide clinical decision-making and improve patient outcomes.

## Discussion

Gastric cancer is still a serious global public health risk (25). Despite the advent of new treatment modalities, recurrence and metastasis continue to be the cause of death in patients with gastric cancer (26). It’s learnt from a previous study that stress from perioperative events can lead to the spread and metastasis of gastric cancer even in patients undergoing radical gastric cancer surgery (27). In addition, oxidative stress contributes an equally crucial factor in the progression of gastric cancer (28, 29). In this study, the predictive value of SOS on the prognosis of patients undergoing gastric cancer surgery was investigated for the first time.

Relying solely on a singular oxidative stress-related biochemical index for prognostic prediction in gastric cancer patients undergoing surgical treatment presents inherent limitations. To overcome this, our study employed a multi-faceted approach. Utilizing Kaplan-Meier survival analysis, potential oxidative stress-related biochemical indices linked to prognosis were identified. Subsequently, employing the LASSO COX regression, we amalgamated these indices to formulate the SOS formula. The study robustly substantiates the efficacy of SOS in prognosticating outcomes in gastric cancer patients undergoing surgical treatment, a validation reinforced by its applicability in assessing OS within the validation cohorts. Significantly, subgroup analyses focused on the nature of surgical resection, distinguishing between R0 and Non-R0 subgroups. Within the R0 subgroup, patients with elevated SOS exhibited an inferior prognosis compared to their low SOS counterparts. However, in the Non-R0 subgroup, the predictive value of SOS for both PFS and OS did not achieve statistical significance. This observed trend may be attributed to the relatively diminutive sample size within the Non-R0 group and the heterogeneity introduced by the inclusion of palliative resections and gastrointestinal reconstructions. Furthermore, the impact of tumor load reduction on host oxidative stress levels within the Non-R0 subgroup is acknowledged as a potential confounding factor.

Studies addressing the prognostic impact of patients’ oxidative stress levels have been conducted in previous clinical studies. For example, Kaiming Zhang et al, used a prognostic model including SOS to predict the prognosis of breast cancer patients treated with surgery (30). Yinghao Cao et al. followed Kaiming Zhang et al’s study and constructed oxidative stress-related indicators for colorectal cancer in the same way, and used the oxidative stress indicator CIOSS to build a prognostic model to predict the prognosis of patients undergoing surgery for colorectal cancer (31).

ALB is also an important substance in the body, which not only responds to the inflammatory status of the organism, but also has its own antioxidant effect, and because of the free thiol group of albumin Cys34, it is able to engage reactive oxygen species in redox reactions (32, 33). The isoenzyme in lactate dehydrogenase: lactate dehydrogenase A. LDHA exist more abundantly in tumor tissues than in normal tissues. LDHA acts as a glycolytic gene prompting the conversion of pyruvate to lactate (34). Studies have shown that the production of LHDA can be reduced by short interfering RNA (siLDHA), and LDHA reduction alleviates oxidative stress in cells due to the conversion of pyruvate to lactate (35, 36). CRE is produced by muscle metabolism and eliminated via the kidneys, and elevated levels of oxidative stress lead to impaired CRE elimination (37). Cisplatin is a more than commonly used anti-cancer drug in the treatment of gastric cancer, but its side effect renal damage also limits clinical work. It was found that hesperidin attenuates oxidative stress induced by cisplatin by activating the NF2 signaling pathway (38). Therefore CRE can be used as a laboratory indicator to respond to the level of oxidative stress in the body. Uric acid is the product of purines catalyzed twice by xanthine dehydrogenase and xanthine oxidase, the latter using molecular oxygen as an electron acceptor to generate reactive oxygen products such as superoxide anion (39, 40). Uric acid has been proven to be an antioxidant substance. It can be excreted not only through the kidneys but also through the intestine (41, 42). Studies have demonstrated that UA and its oxidation product allantoin are presented as gastrointestinal contents, with the highest content of UA and allantoin in the duodenum and jejunum, and that activation of NF2 promotes the synthesis and secretion of UA, which can play an antioxidant role in the gastrointestinal tract (43).

Yet, there are still unavoidable limitations of this study for the following reasons. First, the relationship between the laboratory markers that make up SOS and oxidative stress and the mechanisms are unclear. Second, selection bias cannot be ruled out. Third, SOS is not a tumor-specific marker, which may also have significance for the diagnosis or prognosis of other diseases or cancers. Fourth, this study was conducted in a single-center retrospective method, and large-scale multicenter prospective studies are still required.

## Conclusion

SOS is thought to be a significant predictor of survival and prognosis for individuals with gastric cancer undergoing surgical treatment. High SOS levels in patients typically result in less favorable clinical outcomes.

## Data availability statement

The original contributions presented in the study are included in the article/supplementary material. Further inquiries can be directed to the corresponding author.

## Ethics statement

This study was approved by the ethics committee of Harbin Medical University Cancer Hospital. All patients provided written informed consent before the study. (Ethics number: 2019-57-IIT).

## Author contributions

XW: Writing – original draft. LZ: Data curation, Writing – review & editing.
